# Design and Modeling of a Novel Tripteron-Inspired Triaxial Parallel Compliant Manipulator with Compact Structure

**DOI:** 10.3390/mi13050678

**Published:** 2022-04-27

**Authors:** Yanlin Xie, Yangmin Li, Chifai Cheung

**Affiliations:** State Key Laboratory of Ultra-precision Machining Technology, Department of Industrial and Systems Engineering, The Hong Kong Polytechnic University, Hung Hom, Kowloon, Hong Kong; yanlin.xie@connect.polyu.hk (Y.X.); benny.cheung@polyu.edu.hk (C.C.)

**Keywords:** tripteron, triaxial, parallel-kinematic, compliant manipulator, compact structure

## Abstract

Compliant mechanisms are popular to the applications of micro/nanoscale manipulations. This paper proposes a novel triaxial parallel-kinematic compliant manipulator inspired by the Tripteron mechanism. Compared to most conventional triaxial compliant mechanisms, the proposed manipulator has the merits of structure compactness and being free of assembly error due to its unique configuration and the utilize of 3D printing technology. The compliance matrix modeling method is employed to determine the input stiffness of the compliant manipulator, and it is verified by finite-element analysis (FEA). Results show that the deviations between simulation works and the derived analytical models are in an acceptable range. The simulation results also reveal that the compliant manipulator can achieve a 16 μm × 16 μm × 16 μm cubic workspace. In this motion range, the observed maximum stress is much lower than the yield strength of the material. Moreover, the dynamic characteristics of the manipulator are investigated via the simulations as well.

## 1. Introduction

Piezo-driven compliant mechanisms have received increasing attention in both academic and industrial communities, due to their merits of micro/nanoscale resolution and fast response. They have been widely applied to a variety of fields such as atomic force microscopy [1], precise assembly [2], cell micro-injection [3], micro/nanoscratching [4] and micro electromechanical systems [5]. The compliant mechanisms transmit motion based on the deformation of flexure hinges, which are different from conventional mechanisms on the basis of rigid links and gears. They are capable of overcoming the disadvantages of traditional mechanisms including the backlash and assembly error [6,7]. The piezoelectric actuator (PEA) is a normally-used driving component in compliant mechanisms owing to its high resolution, rapid response and ease of compact size [8,9,10]. With all outstanding superiorities as mentioned, the piezo-driven compliant mechanism has been one of the promising choices for precision manipulators [11,12,13].

The translational XYZ compliant mechanism is a significant manipulator in some applications such as the atomic force microscopy and micro-injection. They can be commonly divided into serial mechanisms and parallel mechanisms based on the kinematic principle. For the serial mechanisms, each of the axes works independently and is easy to control, since they do not have coupling motions between axes. Wadikhaye et al. [14] designed a serial-kinematic XYZ positioner with rapid response and compact structure for the atomic force microscopy. The positioner has a reachable motion range of 8 μm × 6 μm × 2 μm with the frequencies of 10, 7.5 and 64 kHz along the *x*, *y* and *z*-axis, respectively. Kenton et al. [15] proposed a serial-kinematic triaxial compliant mechanism. A positioning range of 9 μm × 9 μm × 1 μm is achieved with the fabricated prototype. The experimental results also demonstrate that the mechanism has the resonant frequencies of 24.2, 6 and 70 kHz along the *x*, *y* and *z*-axis, respectively. A three-axis serial-kinematic positioning device driven by piezoelectric actuators was developed for fast tool servo application [16]. The natural frequencies of the positioner along the *x*, *y* and *z*-axis were measured at 1.06, 0.65 and 0.54 kHz, respectively. However, the accumulation of error is inevitable for the serial structure, and it is at the sacrifice of the natural frequency because the serial connection increases the mass of motion parts of other axes, resulting in different resonant frequencies among axes [17,18].

On the contrary, the motion platform is directly connected with all axes in the parallel mechanisms, so that the cumulative error and motion mass increase can be avoided. Many parallel XYZ compliant mechanisms have been developed due to their virtues of high precision and high resonant frequency. Li et al. [19] proposed a parallel XYZ compliant mechanism with good motion decoupling properties in both the input end and the output platform. In the design, the compound parallelogram flexures were adopted to realize the total decoupling. The finite element analysis (FEA) simulation results show that it has identical dynamic performance in all axes, and experimental results demonstrate that the developed mechanism can achieve submicron accuracy. By introducing the biaxial right circular flexure hinges, Zhu et al. [20] developed a parallel triaxial translational monolithic compliant mechanism, which aims to achieve high bandwidth, high stiffness and high compactness. For the sake of enlarging the mechanism workspace, a 3-DoF XYZ precision positioner based on the modified differential lever amplifier was proposed in the literature of [21].Nonetheless, the base frames in the aforementioned works were not clearly displayed and the overall body sizes were difficult to evaluate. In the literature of [22], a complete parallel XYZ micromanipulator with the base frame was presented. Tang et al. [23] also conceived and designed a decoupled XYZ flexure parallel mechanism mounted on the base frame. Hao et al. [24] developed a 3 DoF translational compliant manipulator with three XY orthogonally-placed flexure mechanisms fixed on the base frame. With the same research group, spatial double four-beam modules were employed in the design of a parallel modular XYZ compliant mechanism referring to a 3-PPPR (P: prismatic, R: revolute) kinematic principle. The developed mechanism also took into account the base frame in the design [25]. In addition, Awtar et al. [26,27] proposed a parallel triaxial translational flexure mechanism mounted on the base with a travel range of 10 mm × 10 mm × 10 mm. Good decoupled translational motions among axes and high stiffness in the rotational motions were observed. However, the body sizes of some of the above-mentioned manipulators are bulky, while the base frame structures are considered. Moreover, the assembly errors between amplifier mechanisms/guidance mechanisms and base frames in these studies are difficult to be avoided.

The kinematics-based approach, the building blocks approach and the topology optimization approach [28,29,30] are three classical compliant mechanisms designing methods. On the basis of the kinematics-based approach, a novel mechanism inspired by the Tripteron [31] is proposed in this paper to solve the aforementioned problems, which may provide an alternative way to construct the parallel XYZ compliant manipulator. 3D printing technology is expected to monolithically fabricate the proposed manipulator for eliminating assembly error and realizing structure compactness. The rest of this paper is structured as follows. Section 2 illustrates the overall configuration and working principle of the parallel XYZ manipulator. In Section 3, the compliance matrix modeling method is utilized to analyse the stiffness/compliance of the manipulator. FEA simulation works are conducted to verify the theoretical analyses in Section 4. Section 5 draws a brief conclusion of this work.

## 2. Mechanism Design

**Figure 1:**
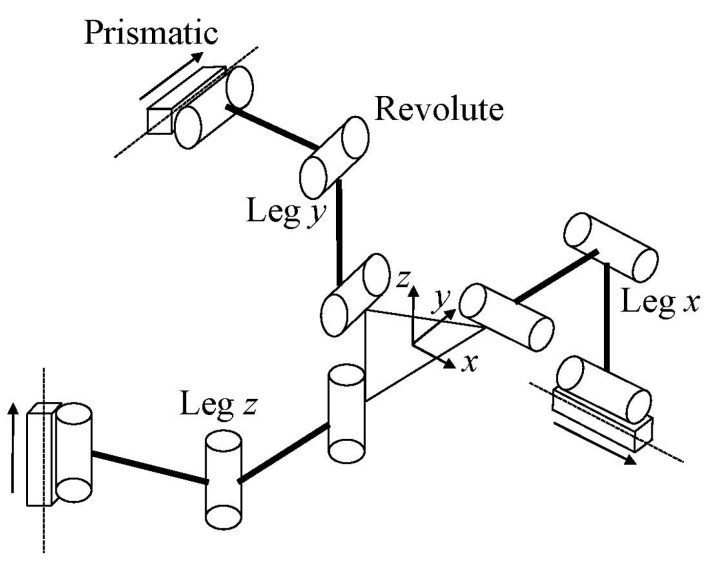
The kinematic diagram of Tripteron.

As shown in Figure 1, the Tripteron is a triaxial translational parallel mechanism [31,32]. It is composed of an orthogonally arranged base frame, three kinematically identical legs and an end-effector. Referring to the kinematics model of the Tripteron, the leg has three revolute joints on its own and a prismatic joint connected with the base frame. When the prismatic joint is driven by the linear motor, the end-effector can achieve consistent translational motion along the driving axis. As a result, the Tripteron has three translational motions in the Cartesian coordinate. Investigation into compliant Triteron is still scarce, although a compliant Tripteron has been presented previously, and the kinematostatic model has been established in the literature of [33]. In the reported literature, cruciform hinges were employed, whereas the right circular flexure hinges and beam flexure hinges will be adopted in the current paper. Furthermore, the reported compliant Tripteron is not a monolithic mechanism, which is different from the proposed mechanism in this paper.

**Figure 2:**
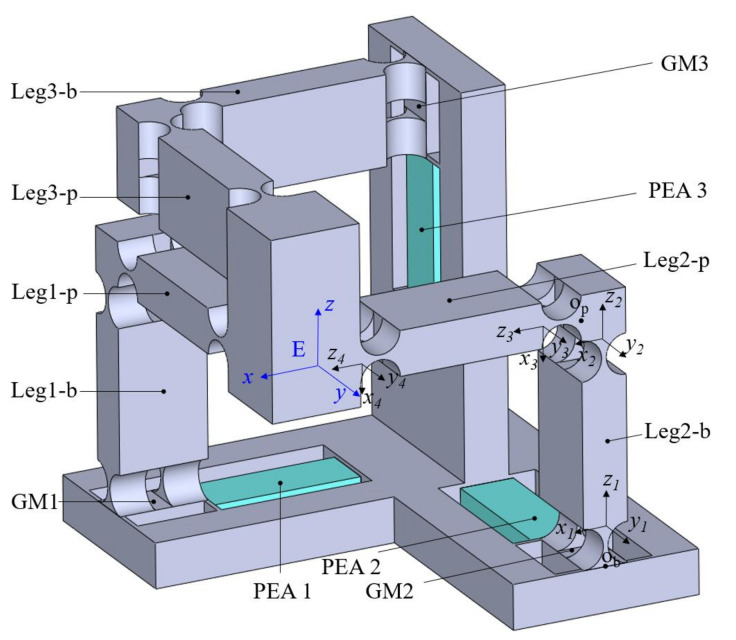
Mechanical structure of the proposed triaxial compliant manipulator.

As enumerated above, a new translational XYZ parallel compliant mechanism based on the Tripteron is devised as shown in Figure 2. It consists of a base, three kinematically identical legs and a motion platform. The base structure is a guidance mechanism (GM) embedded with a PEA along each axis of the Cartesian coordinates. The leg is a rectangular-shaped compliant mechanism. The leg is worked as a driving unit while it moves toward the elongation direction of the directly connected PEA, otherwise it works as a passive guidance unit along the other two axes. For the convenience of analyses in the following sections, each leg is further subdivided into leg-p which is directly connected with the platform and leg-b which is directly connected with the base frame.

The right circular flexure hinge and beam flexure hinge are commonly used flexure hinges. The right circular flexure hinge has the merits of little axis drift, high-precision and high transversal stiffness. The beam flexure hinge can distribute the deformation in the whole beam and avoid the concentration of stress, which provides a larger displacement of deformation without failure. As a result, the GM 1 is constructed with beam flexure hinges, which mainly aims to provide large output displacement. On the other hand, the guidance mechanism has four parallel connected beam flexure hinges, which can avoid the coupled motion caused by other driving units including the transverse motion and twisted deformation. This is significant for protecting the PEAs from being damaged, since they are sensitive to the tangential force and bending deformation. In contrast, the right circular flexure hinges are used in the design of the legs, so that the high lateral stiffness can alleviate the deformation when they are working as driving units, and the compliance can reduce the constraint stiffness when they are working as passive guidance units.

The working principle of the proposed 3-DoF compliant mechanism can be illustrated as follows. The PEA 1 elongates when the voltage is exerted on it, driving the GM 1 and corresponding connected leg toward the positive *x*-axis, and thus the motion platform moves toward positive *x*-axis accordingly. Based on the same principle, the motions along *y*-axis and *z*-axis can be achieved when the voltages are applied at PEA 2 and PEA 3. As a result, a compliant mechanism with translational 3-DoF is developed.

## 3. Modeling and Analysis

### 3.1. Modeling Method

There are many modeling methods for analyzing compliant mechanisms as reviewed in the literature of [34,35,36], including Castigliano’s second theorem, elastic beam theory, compliance matrix modeling (CMM) method, finite element method, pseudo-rigid-body (PRB) method, the chained beam constraint model and the 3-D dynamic stiffness model. Both merits and shortcomings were comprehensively reported. In this paper, the PRB method [37] and CMM method [38,39] are mainly discussed. The PRB method only takes account of the compliance of flexure hinges along the working direction, so that it is not able to achieve a complete compliance analysis. Moreover, it involves loop closure theory, virtual work principle and Lagrange’s equation, which is complex for complicated mechanisms. On the contrary, the CMM method on the basis of Hooke’s law can establish 6-DoF compliance in the space of flexure hinges with high accuracy. Furthermore, it has high calculation efficiency due to the effective operation of matrix with the help of a computer. As a result, the compliance/stiffness of the proposed compliant mechanism in this paper is analyzed based on the compliance matrix modeling method.

**Figure 3:**
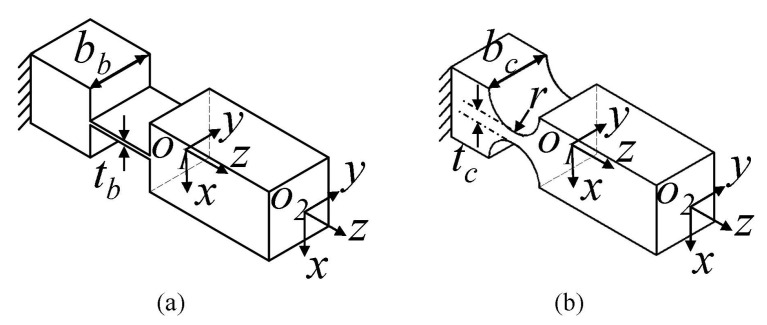
The beam flexure hinge (**a**) and right circular flexure hinge (**b**).

As shown in Figure 3, the compliance matrix in its local coordinate can be expressed as:

(1) $$C_{f}=\left[\begin{array}{cccccc} c_{1} & 0 & 0 & 0 & c_{3} & 0\\ 0 & c_{2} & 0 & -c_{4} & 0 & 0\\ 0 & 0 & c_{5} & 0 & 0 & 0\\ 0 & -c_{4} & 0 & c_{6} & 0 & 0\\ c_{3} & 0 & 0 & 0 & c_{7} & 0\\ 0 & 0 & 0 & 0 & 0 & c_{8} \end{array}\right],$$

where the specific values of compliance factors of different flexure hinges are listed in the literature [40]. Equation (2) shows the conversion of the compliance matrix from its local coordinate to a new coordinate frame.

(2) $$C_{2}=T_{1}^{2}C_{1}{\left(T_{1}^{2}\right)}^{T},$$

where $C_{1}=C_{f}$ is the compliance matrix of the flexure hinge with respect to the fixed-end. The coordinate transformation matrices $T_{1}^{2}$ include the rotational matrix and the translational matrix. ${\bar{R}}_{x}$, ${\bar{R}}_{y}$, ${\bar{R}}_{z}$ are the rotational matrices around the *x*, *y* and *z*-axis, and they can be written as

(3) $${\bar{R}}_{x}\left(\gamma \right)=\left(\begin{array}{cc} R_{x}\left(\gamma \right) & 0\\ 0 & R_{x}\left(\gamma \right) \end{array}\right),$$

(4) $${\bar{R}}_{y}\left(\beta \right)=\left(\begin{array}{cc} R_{y}\left(\beta \right) & 0\\ 0 & R_{y}\left(\beta \right) \end{array}\right),$$

(5) $${\bar{R}}_{z}\left(\alpha \right)=\left(\begin{array}{cc} R_{z}\left(\alpha \right) & 0\\ 0 & R_{z}\left(\alpha \right) \end{array}\right),$$

where $R_{x}\left(\gamma \right)$, $R_{y}\left(\beta \right)$ and $R_{z}\left(\alpha \right)$ denote the rotation around the corresponding axis. The translational matrix $\bar{q}=\left(q_{x},q_{y},q_{z}\right)$ can realize the translation of the compliance matrix, and it is given by

(6) $$\bar{q}\left(q_{x},q_{y},q_{z}\right)=\left(\begin{array}{cc} I & \hat{q}\\ 0 & I \end{array}\right),$$

where $\hat{q}$ represents the outer product for a vector $q=(q_{x},q_{y},q_{z})$, and it can be derived as Equation (7), in which *q* is the coordinate of the new coordinate system relative to the transferred coordinate frame. The identity matrix *I* is described by Equation (8).

(7) $$\hat{q}=\left(\begin{array}{ccc} 0 & -q_{z} & q_{y}\\ q_{z} & 0 & -q_{x}\\ -q_{y} & q_{x} & 0 \end{array}\right),$$

(8) $$I=\left(\begin{array}{ccc} 1 & 0 & 0\\ 0 & 1 & 0\\ 0 & 0 & 1 \end{array}\right).$$

### 3.2. Input Stiffness of the Proposed Mechanism

For the purpose of determining the compliance/stiffness of the compliant mechanism, the mechanism has been divided into two parts for easy analysis. The compliances/stiffnesses of the guidance mechanisms on the base and legs connected motion platform and guidance mechanisms are separately analyzed first, and are then added together based on the principle of parallel and series connection.

**Figure 4:**
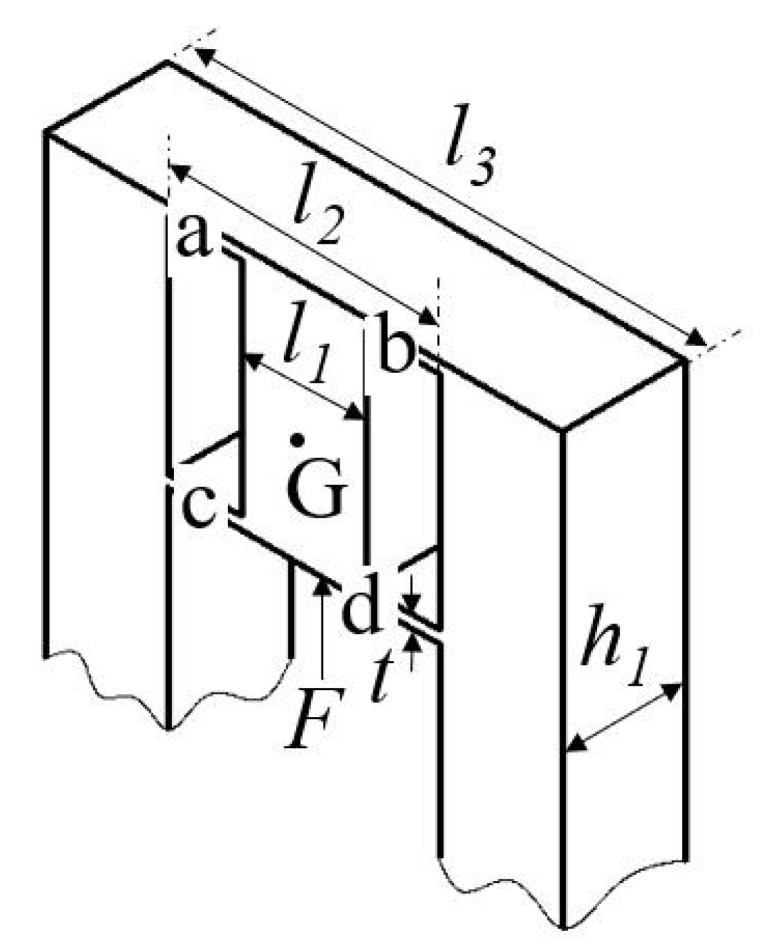
The guidance mechanism in the base frame.

As shown in Figure 4, the GM 3 on the base along the *z*-axis is picked out for analysis. On the basis of the compliance matrix modeling method, the compliance of hinge *a* can be generated as

(9) $$C_{a}^{G}=T_{a}^{G}C_{a}{\left(T_{a}^{G}\right)}^{T}.$$

Due to flexure hinges *b* and *a* are symmetric around the *x*-axis, the compliance of hinge *b* can be derived as

(10) $$C_{b}^{G}={\bar{R}}_{x}\left(\pi \right)C_{a}^{G}{\left({\bar{R}}_{x}\left(\pi \right)\right)}^{T}.$$

The above mentioned principle is also applicable to flexure hinges *c* and *d*, and thus their compliance matrices can be obtained as

(11) $$C_{c}^{G}=T_{c}^{G}C_{c}{\left(T_{c}^{G}\right)}^{T},$$

(12) $$C_{d}^{G}={\bar{R}}_{x}\left(\pi \right)C_{c}^{G}{\left({\bar{R}}_{x}\left(\pi \right)\right)}^{T}.$$

Owing to the four flexure hinges being parallel connected with the motion platform *G*, the vertical compliance $C_{v}^{G}$ and vertical stiffness $K_{v}^{G}$ can be determined by

(13) $$C_{v}^{G}={\left({\left(C_{a}^{G}\right)}^{-1}+{\left(C_{b}^{G}\right)}^{-1}+{\left(C_{c}^{G}\right)}^{-1}+{\left(C_{d}^{G}\right)}^{-1}\right)}^{-1},$$

(14) $$K_{v}^{G}={\left(C_{v}^{G}\right)}^{-1}.$$

**Figure 5:**
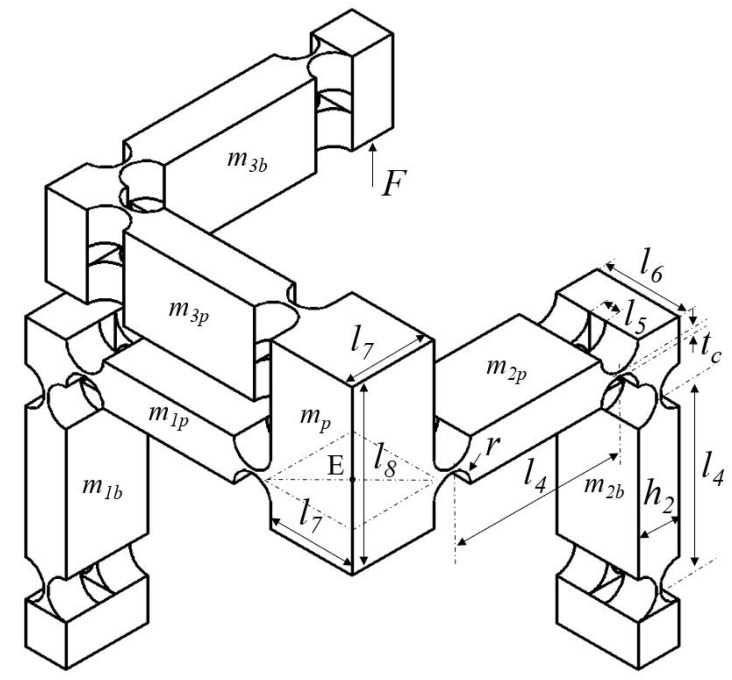
Schematic of the motion platform and three legs.

Following the previous assumption that the leg connected with the *z*-axis is the driving unit as shown in Figure 5, and the leg1-p and leg2-p are worked as guidance mechanisms, referring to Figure 2, leg2 is selected for the analysis. Considering that two layers of flexure hinges parallel connect the motion platform and leg2-b, the compliance of point *E* with respect to the point Op can be derived as

(15) $$C_{E}^{p}={\left({\left(T_{3}^{E}C_{3}{\left(T_{3}^{E}\right)}^{T}+T_{4}^{E}C_{4}{\left(T_{4}^{3}\right)}^{T}\right)}^{-1}+{\left(T_{3}^{E}C_{3}{\left(T_{3}^{E}\right)}^{T}+T_{4}^{E}C_{4}{\left(T_{4}^{3}\right)}^{T}\right)}^{-1}\right)}^{.}$$

Due to leg1-p and leg2-p being parallel connected with the motion platform, and having circular symmetry of $90^{\circ }$ around the *z*-axis, the compliance $C_{G}^{1p2p}$ and the stiffness $K_{G}^{1p2p}$ can be derived as

(16) $$C_{G}^{1p2p}={\left(K_{G}^{1p2p}\right)}^{-1}={\left({\left(C_{E}^{p}\right)}^{-1}+{\left(T_{1p}^{2p}C_{E}^{p}{\left(T_{1p}^{2p}\right)}^{T}\right)}^{-1}\right)}^{-1},$$

where $T_{1p}^{2p}$ is the transformation matrix transferring from leg1-p to leg2-p, and it can be written as

(17) $$T_{1p}^{2p}=\left(\begin{array}{cc} R_{z}\left(-\pi /2\right) & 0\\ 0 & R_{z}\left(-\pi /2\right) \end{array}\right).$$

As a result, the total input stiffness along the *z*-axis of the compliant mechanism $K_{z}$ can be obtained as the following equation since the GM 3 and guidance mechanism 1*p*2*p* are parallel connected with the motion platform

(18) $$K_{z}=K_{v}^{G}+K_{G}^{1p2p}.$$

According to the parallel kinematics of the GM 3 and guidance mechanism 1*p*2*p*, the output stiffness should be equal to the input stiffness. However, the distortion of the cantilever structure of the driving leg will reduce the output stiffness to some extent. The stiffnesses/compliances of the compliant mechanisms along other two axes can also be determined based on the same principle.

## 4. Model Validation and Performance Evaluation via FEA

Table 1 shows the structural parameters of the developed parallel triaxial compliant mechanism. 3D printing technology is expected to fabricate the mechanism, due to its capability of manufacturing monolithic complex structure with high dimension accuracy on the basis of the photosensitive resin material [41,42,43]. The detail physical properties of resin are: Young’s modulus E=2.2 GPa, Poisson’s ratio μ = 0.394, density $\rho =1.18\times 10^{3}$ kg/m${}^{3}$, and yield strength $\delta_{s}=50$ MPa. According to the above mentioned parameters, FEA simulations are conducted to validate the established theoretical model and to evaluate the performance of the proposed compliant mechanism with the help of commercial software ANSYS.

**Figure 6:**
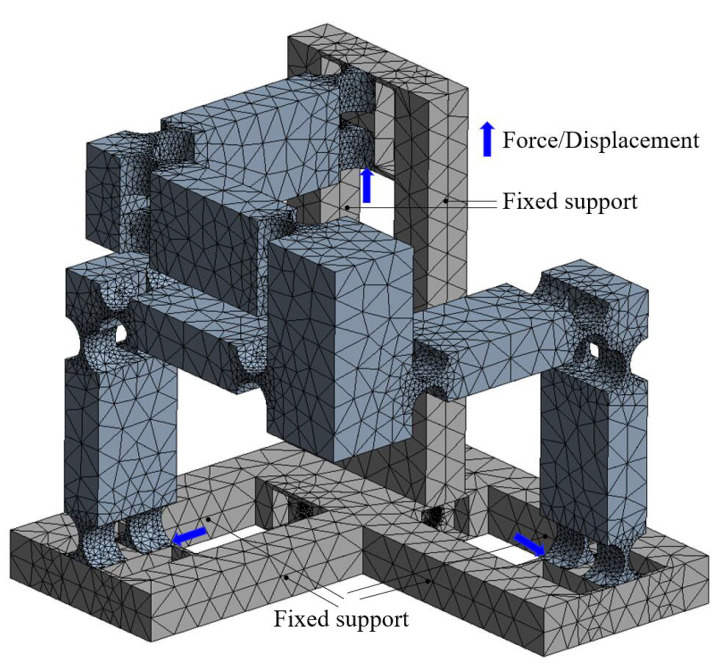
Details of the FEA model.

**Figure 7:**
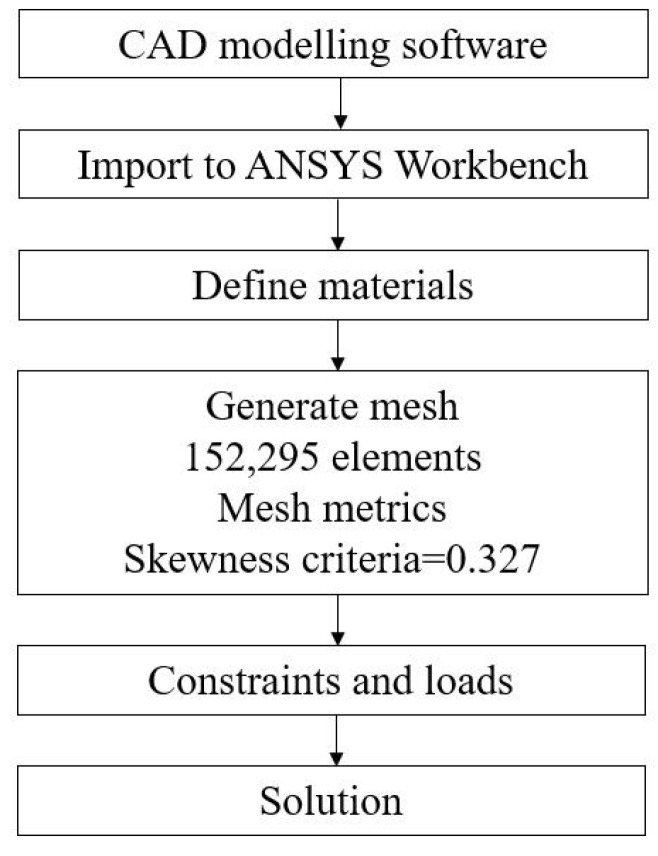
Flowchart of the FEA simulation.

The details of the FEA model are shown in Figure 6, including the constraints, loads, etc. In addition, a flowchart of conducting the FEA simulations is also given in Figure 7. Starting from building the 3-D model of the compliant manipulator via the CAD modelling software, the model is then imported to the ANSYS workbench to perform the simulation analysis. During the simulation, the material of the mechanism was defined according to the aforementioned details of the material properties. Following is the mesh generation. The mesh has 152,295 elements. The skewness criteria mesh metrics is adopted to evaluate the mesh quality and the average value is 0.327, which shows that it has a very good quality. After that, the constraints and loads are defined in the model, and the simulation results can be obtained after the calculation.

**Figure 8:**
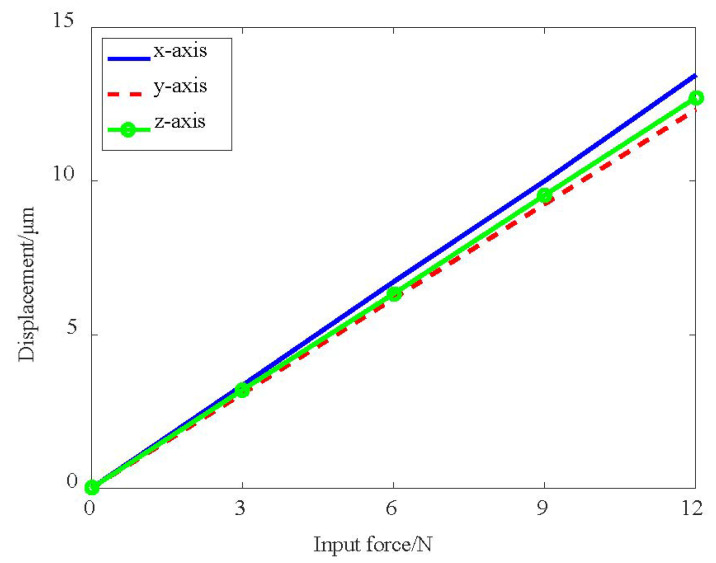
The correlation between input force and output displacement.

The correlation between the output displacement of the moving platform and the input force is shown in Figure 8. In the figure, three almost coinciding straight lines can be seen, which means that the three axes have a nearly identical linear relationship. The input stiffness is derived by dividing the input force with the output displacement of the moving platform, which is the reciprocal of the slope of the line in Figure 8. The comparison between analytical models and simulation results are given in Table 2. The deviations are mainly induced by the deformation of the driving legs due to the cantilever structure, which can be further explained in the next discussion of motion loss.

**Figure 9:**
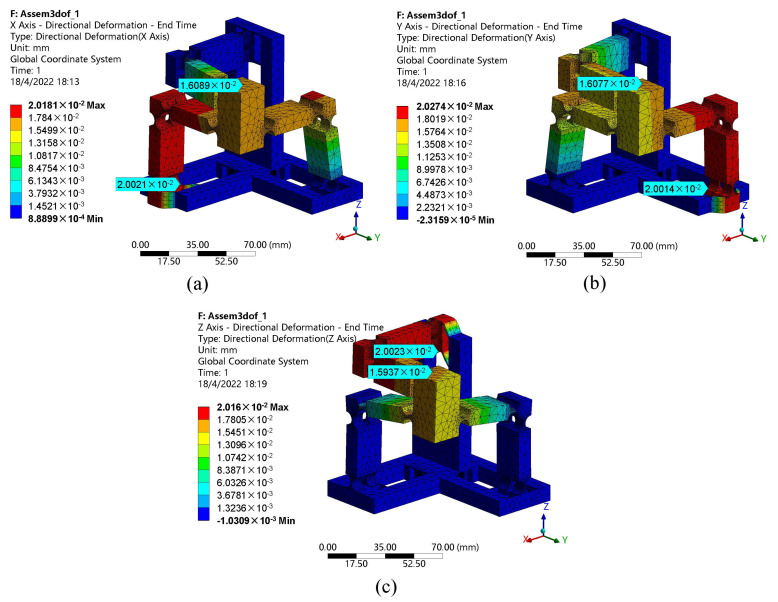
The corresponding output displacement of the motion platform with a 20 μm input on different axes: (**a**) *x*-axis, (**b**) *y*-axis, (**c**) *z*-axis.

Figure 9 illustrates the output displacement of the motion platform with a 20 μm input displacement on each axis. One can observe that the corresponding output displacement of the motion platform is 16 μm with the same direction as the driving axis, which has a motion loss of 20%. The motion loss is mainly caused by the constraint of the passive legs and the deformation of the driving leg which is a cantilever structure. In the case of 20 μm input, the deflection of the cantilever beam can be determined by $\omega =Fl^{3}/3EI$ and it is equal to 1 μm, which is still smaller than the motion loss of 4 μm. This deviation may be further linked to the following two reasons. Firstly, the right circular flexure hinge on the leg may increase the distortion of the structure. For the next reason is that the leg is fixed on the GM, and the GM is connected with the base frame using beam flexure hinges, which may also induce the distortion.

The decoupling property of the compliant mechanism is depicted in Table 3. When the motion platform is driven toward *x*-axis with a displacement of 16, the cross-axis motions along the *y* and *z* axes are 1.4 and −0.84 μm, respectively. A major motion along the *y*-axis induces the parasitic motions of 2.82 and −0.62 μm along the *x* and *z* axes. Similarly, the couple motions along the *x* and *y* axes caused by the output displacement of 16 μm in the *z*-axis are −0.96 and 1.41 μm. The results reveal that the decoupling properties among all axes of the proposed mechanism are not as good as they should be, and it may be linked to the distortion of the driving legs.

**Figure 10:**
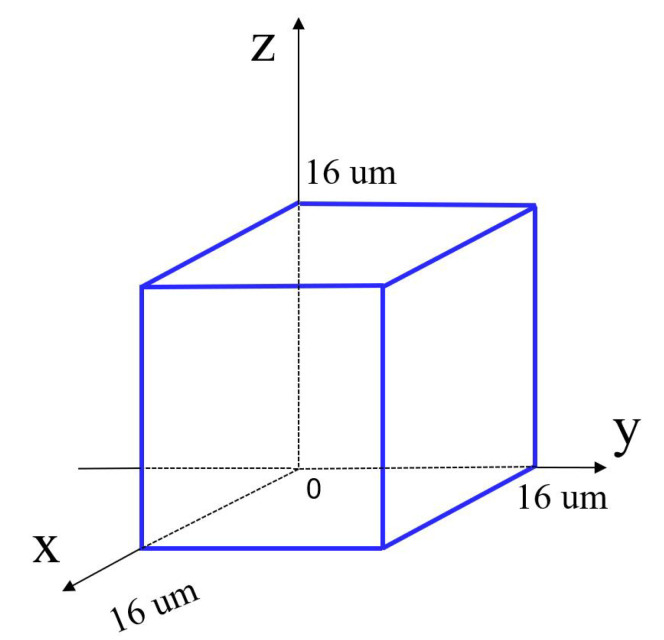
The reachable workspace of the proposed triaxial compliant manipulator.

**Figure 11:**
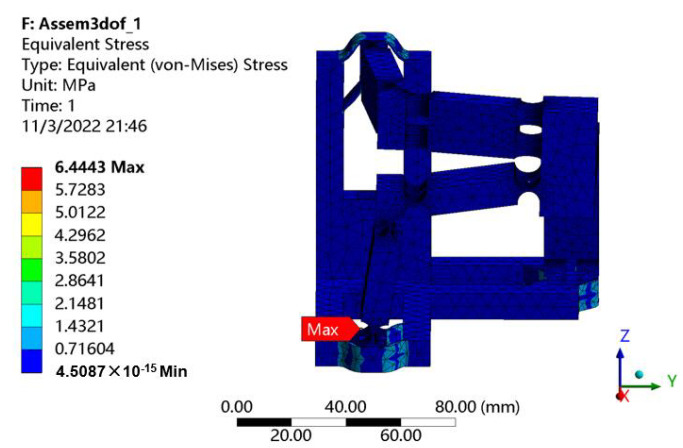
Stress distribution.

With the consideration of the maximum stroke of the selected PEA, the workspace of the mechanism can be obtained as shown in Figure 10. The reachable workspace is a cube with 16 μm along each axis. In addition, the maximal stress of 6.44 MPa occurred in the beam flexure hinges can be observed from Figure 11 when the maximum stroke of 20 μm of the PEA is exerted on the input end. It is much lower than the yield strength 50 MPa, indicating that the proposed mechanism is capable of working normally without failure.

**Figure 12:**
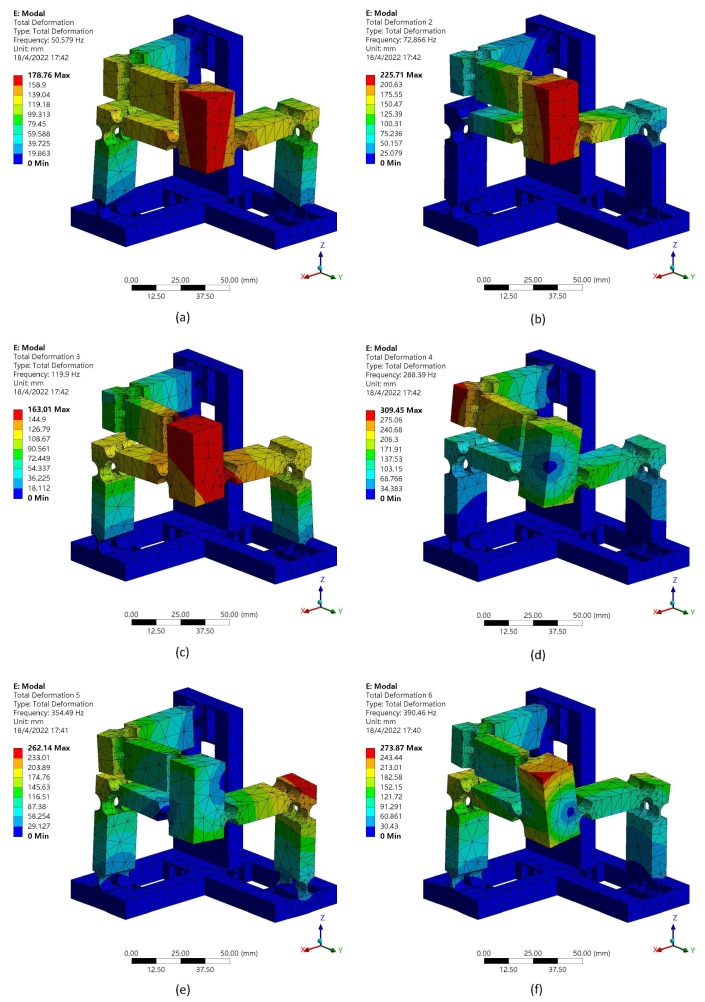
First six mode shapes: (**a**) mode 1, (**b**) mode 2, (**c**) mode 3, (**d**) mode 4, (**e**) mode 5, (**f**) mode 6.

The dynamic performances are also studied with the FEA methodology. The first six mode shapes are shown in Figure 12. The first mode has a frequency of 50.58 Hz. The second and third modes of the proposed compliant mechanism have the frequencies of 72.87 Hz and 119.9 Hz, respectively. The corresponding value of the fourth, fifth, sixth modes are 288.39 Hz, 354.49 Hz and 390.46 Hz.

It can be observed that the first three resonance frequencies are different and the reasons are as follows. According to the working principle of the manipulator, when the motion platform moves toward *x*-axis, $m_{1b}$, $m_{1p}$ and $m_{2p}$ share the same displacement of the motion platform. However, $m_{2b}$ and $m_{3p}$ only have half of the displacement. When the motion platform moves toward *y*-axis, $m_{1p}$, $m_{3p}$, $m_{2b}$ and $m_{2p}$ share the same displacement of the motion platform. However, $m_{1b}$ and $m_{3b}$ only have half of the displacement. When the motion platform moves toward the *z*-axis, $m_{3b}$, $m_{3p}$ share the same displacement of the motion platform. However, $m_{1p}$ and $m_{2p}$ only have half of the displacement. As a result, the equivalent masses in different directions are different, with the consideration of the same equivalent stiffness as given in Table 2, and thus the vibration modes have different resonance frequency values.

## 5. Conclusions

A novel triaxial parallel-kinematic compliant manipulator inspired by the Tripteron mechanism is proposed and analyzed in this paper. The manipulator is composed of a base structure, three legs and a motion platform. Unlike the design of most conventional triaxial compliant mechanisms, the base frames of the mechanisms were ignored. The proposed compliant manipulator has taken it into consideration in the design. Due to the unique configuration of the proposed manipulator, it is able to realize the triaxial translational motions with a compact structure. The assembly error can also be avoided by fabricating the mechanism monolithically with the help of 3D printing technology. The stiffness/compliance model of the mechanism is established based on the compliance matrix modeling method and verified by FEA simulations. The deviations between them are in a reasonable range. A cubic workspace with 16 μm along each axis is observed with a maximum stress much lower than the yield strength, which means the manipulator is in safe working condition, although the decoupling performance is not as good as expected. In addition, the dynamic performances of the manipulator are also indicated via the simulations. In the future research, 3D printing technology is expected to fabricate the prototype to experimentally study the comprehensive performances of the proposed manipulator.

## Figures and Tables

**Table 1:** Dimensional parameters of the proposed triaxial compliant manipulator.

GM 3		Legs and the Motion Platform	
**Symbol**	**Value (mm)**	**Symbol**	**Value (mm)**
$l_{1}$	10	$l_{4}$	41
$l_{2}$	22	$l_{5}$	4
$l_{3}$	42	$l_{6}$	20
$h_{1}$	10	$h_{2}$	10
*t*	0.6	$t_{c}$	1
		*r*	4.5
		$l_{7}$	20
		$l_{8}$	40

**Table 2:** Comparison results of input stiffness.

Methods	*x*-Axis Input/Output Stiffness (N/µm)	*y*-Axis Input/Output Stiffness (N/µm)	*z*-Axis Input/Output Stiffness (N/µm)
Analytical model	0.82	0.82	0.82
FEA	0.89/0.69	0.97/0.70	0.94/0.72
Error	−18%/19%	−15%/17%	−13%/14%

**Table 3:** Performance of the proposed triaxial compliant manipulator.

Input Displacement (µm)			Output Displacement (µm)		
**x**	**y**	**z**	**x**	**y**	**z**
20	-	-	16	1.40	−0.84
-	20	-	2.82	16	−0.62
-	-	20	−0.96	1.41	16

## Data Availability

Not applicable.
